# Bridging the silver–digital divide: how digital literacy shapes diverse healthcare utilisation among China’s older adults—a cross-sectional study in seven Chinese cities

**DOI:** 10.3389/fpubh.2025.1577231

**Published:** 2025-11-12

**Authors:** Chi Zhang, Jiayu Hou, Hengyuan Zhang

**Affiliations:** 1 School of Philosophy and Sociology, Lanzhou University, Lanzhou, China; 2 School of Public Policy and Administration, Xi’an Jiaotong University, Xi’an, China

**Keywords:** digital literacy, healthcare consumption, older adults, social support, technology acceptance

## Abstract

**Background:**

As of December 2023, China’s population aged 60 and above reached 296.97 million, accounting for 21.1% of the total population. The convergence of an aging society and a digital society presents significant challenges for older adults, particularly in terms of digital inclusion and access to healthcare. This study investigates the impact of digital literacy on the consumption of healthcare services among older adults in China and explores the underlying mechanisms involving social support and technology acceptance.

**Methods:**

Data from 1,107 valid questionnaires from adults aged 60 and above were analyzed. Digital literacy was assessed using a unidimensional 10-item scale developed for this study. Healthcare service consumption was measured using a validated 8-item scale covering two dimensions: therapeutic and preventive services. Data analysis employed OLS regression, mediation, and moderation models. This study adopted a cross-sectional design with two independent waves of surveys (2019 and 2022) to avoid repeated sampling of the same individuals.

**Results:**

Digital literacy demonstrated a significant positive effect on the consumption of healthcare services among older adults (*p* < 0.01), encompassing both therapeutic and preventive services. Age, urban residence, and education level were also significant positive predictors. Both formal and informal social support were identified as significant mediators in this relationship, with informal support exhibiting a stronger mediating effect. Specifically, digital literacy was positively associated with overall healthcare utilisation (*β* = 0.218, 95% CI: 0.182–0.254, *p* < 0.01), therapeutic services (*β* = 0.182, 95% CI: 0.145–0.219, *p* < 0.01), and preventive services (*β* = 0.265, 95% CI: 0.223–0.307, *p* < 0.01).

**Conclusion:**

Enhancing digital literacy is a crucial strategy for promoting healthcare service utilisation among older adults in an increasingly digitalized society. Policymakers should prioritize improving digital literacy and integrating digital technology into social support systems. Fostering technology acceptance can further amplify these positive effects. This study provides empirical evidence for understanding the role of digital literacy in healthcare consumption and offers valuable insights for policy development. Limitations include the small sample size relative to China’s total older population (*n* = 1,107) and limited geographical coverage (only seven cities), which may restrict the generalisability of findings to remote rural or megacity areas.

## Introduction

As of December 2023, the population of China aged 60 and above is projected to reach 296.97 million, representing 21.1% of the total population. This transition marks the onset of an “aging society” era, which has profoundly impacted the social lives and habits of middle-aged and older adults. Concurrently, the rapid advancements in information technology have led to the emergence of a digital society (1). According to the 53rd Statistical Report on China’s Internet Development, as of December 2023, the number of Internet users in China reached 1.092 billion, with 24.8 million new Internet users compared with December 2022, and the Internet penetration rate reached 77. However, the predominant demographic of Internet users does not consist of older adults, who constitute a substantial segment of the population. Instead, the older adults aged 60 and above constitute the primary non-Internet user demographic, accounting for 39.8% of the overall non-Internet user population (2). It is evident that the convergence of the two waves of “aging” and “digitalisation” has become an inevitable trend of social development. This phenomenon must be given due consideration as it is of significant importance (3).

The wave of population aging is in full swing. Concurrently, the evolution of a digital society has engendered an imperative for individuals to acclimatise to novel technological milieus and lifestyles (4). This dual pressure has been shown to present older adults with a number of challenges in accessing information, participating in social activities, and maintaining social interactions. Such challenges include a lack of digital literacy, digital security issues, lack of social support, and restrictions on leisure activities (2).

Building upon the Technology Acceptance Model and Social Support Theory, this study investigates how older adults access healthcare resources through technology acceptance and formal or informal support systems. While these two frameworks are closely related to the research topic, existing studies such as Grošelj et al. (2) investigation into non-internet usage among seniors have rarely integrated them to systematically explore the complex relationship between digital literacy and multidimensional healthcare utilisation. This research gap has hindered academic exploration of how digital literacy fundamentally influences older adults’ access to diverse medical services, such as telehealth consultations, chronic disease management programs, and preventive healthcare initiatives.

In the face of these challenges, the Chinese government and all sectors of society have implemented a series of measures. Primarily, the government has increased its investment in digital literacy education for the older adults. For instance, in 2020, the General Office of the State Council issued the “Implementation Plan on Effectively Solving the Difficulties of the older adults in the Use of Intelligent Technology,” which recommended the launch of the “Intelligent older adults Assistance” initiative. This initiative aimed to maximize the advantages of intelligent technology to provide services for the older adults in a more convenient, safe and practical way (5). Health consultation services and distance education programs for older adults are provided by government agencies or institutional organizations. These services, distributed through administrative divisions, are delivered either directly or indirectly at community centers or affiliated hospitals, ensuring accessibility for older adults. The services encompass health literacy consultations, targeted disease treatment, and other healthcare support. Furthermore, it has been demonstrated that the digital skills of older adults are enhanced through community education and university-based initiatives, or through the utilisation of social forces, such as volunteer services, to facilitate their integration into the digital society. Despite the implementation of certain measures, the predicament confronting older adults in the digital society persists. For example, the retirement pension for the older adults are not the same in rural and urban areas, which makes the access to healthcare services and information also different; at the same time, for the older adults, they have the same opportunity to access dental care, physical therapy, general medical treatment and hospital treatment, but the actual situation will be different due to the allocation of healthcare resources. Concomitantly, the effective consumption of medical and health services constitutes a pivotal means to ensure the maintenance of their health. The enhancement of the consumption level of healthcare services for older adults has been identified as a prevailing priority of the Chinese government (6). Therefore, an in-depth study of the influence mechanism of digital literacy on the consumption of multidimensional healthcare services by the older adults is of great significance for the development of more effective policies and interventions, as well as providing a theoretical basis and practical guidance for the development of related policies.

To address this critical research gap, this study aims to clarify the theoretical mechanisms through which digital literacy influences older adults’ multidimensional utilisation of medical services via empirical analysis. Theoretically, it integrates two major frameworks to explain the correlation between digital literacy and healthcare service usage, moving beyond isolated analyses of digital non-utilisation or access barriers. This approach provides a holistic understanding of the phenomenon, filling existing research gaps. Practically, it offers actionable insights for policymakers, healthcare providers, and social organizations to design more targeted and equitable measures addressing digital and medical inequalities faced by older adults in an aging digital society.

## Theoretical analysis and research hypotheses

Digital literacy refers to an individual’s ability to use digital technologies, access and process information, communicate and problem solve (7). This concept has evolved in tandem with technological development, thus becoming a significant indicator of the adaptive capacity of members of modern society. The dimensions of digital literacy encompass technical skills, information retrieval, information assessment, information management, communication, content creation, problem solving, ethical and legal considerations, and digital security (8, 9).

The narrow sense of healthcare consumption refers to hospitalization (8), which is often related to hospitals, while the broad sense of healthcare consumption refers to all aspects of healthcare. We operationalised “consumption of HCS” as the number of health-service events (tele-consultations, e-prescription refills, online appointment bookings, use of digital triage tools, and follow-up visits prompted by self-monitoring apps) recorded in the regional e-health platform during the 12-month observation window. The present study is concerned with an investigation into the impact of digital literacy on healthcare consumption among the older adults. The study categorises healthcare consumption into two distinct categories: therapeutic and preventive services (10). Healthcare consumption are measured through self-assessment by the older adults using a five-level scale: 1 = very bad, 2 = bad, 3 = fair, 4 = good, 5 = very good. Anderson’s model is widely regarded as the most classic model in the field of healthcare service consumption research. However, the majority of its research content focuses on the factors affecting the consumption of healthcare services. This study specifies the definition of “digital literacy” and focuses on the research object of older adults, which has a smaller research scope (11). Digital literacy is of pivotal significance in the utilisation of the Internet and digital devices. It is defined by an individual’s capacity to access and employ information judiciously on Internet platforms (12). This study is predicated on the Global Digital Literacy Framework and related research, including that of Terras and Ramsay (12). The study proposes a categorisation of digital literacy into four distinct types: digital device literacy, digital social literacy, digital application literacy and digital security literacy (13). A number of related studies have indicated that residents who use mobile devices to access the Internet are less likely to use healthcare services (14). However, hospitals are the main place where these residents access healthcare services (15). Furthermore, evidence suggests that older adults with lower e-health literacy face challenges in accessing, using, and evaluating online health information. These challenges may have a negative impact on their confidence and attitudes towards health-related information (16). Consequently, older adults with adequate access to information will be more capable of utilising available healthcare resources and managing their health more effectively. In conclusion, digital literacy exerts an indirect or direct influence on the consumption of multidimensional healthcare services by older adults. Therefore, it can be hypothesized:

*H1*: Digital literacy has a significant positive effect on the consumption of healthcare services by the older adults, and the higher the digital literacy, the higher the level of consumption of healthcare services by the older adults.

Formal social support is typically provided by government or organisational agencies, such as online health counseling and distance learning. This formal social support is distinguished by its recurrent nature, frequently underpinned by policy or legal frameworks, and characterised by its personal connection to social organisations. Digital literacy facilitates enhanced access to these resources by older adults for professional and structured support (17, 18). This support has the potential to facilitate greater access to healthcare knowledge and disease management strategies, thereby assisting older adults in optimising their utilisation of healthcare services and enhancing their quality of life (19). Therefore, it can be hypothesized:

*H2*: The formal social support received by older adults moderated by digital literacy to promote the consumption of healthcare services and encourage the utilisation of healthcare services.

Informal social support can be defined as the collection of non-organised social support providers from the older person’s life, including spouses, children, other relatives, neighbours, friends, volunteers, etc. Informal social support is characterised by its interpersonal nature, with relationships being established and maintained on a personal level (20). Digital technologies, including social media and video calling, facilitate communication between older adults and their social networks, as well as provide access to information irrespective of geographical location (21). The provision of immediate, personalised support is of critical importance for older adults, as it facilitates their access to relevant information regarding healthcare services. This support also ensures that the information available to them is more viable and fit for purpose (22). The enhancement of digital literacy among older adults has been demonstrated to facilitate the effective utilisation of these tools, thereby augmenting informal social support and exerting a favorable influence on healthcare service utilisation. Therefore, it can be hypothesized:

*H3*: The informal social support received by older adults moderated by digital literacy to promote the consumption of healthcare services.

In accordance with the Technology Acceptance Model (e.g., UTAUT, Unified Theory of Acceptance and Use of Technology), an individual’s propensity to accept technology has a direct impact on their utilisation of it. Technology acceptance can thus be defined as an individual’s propensity to accept and tendency to use new technology (23). The present study hypothesises that technology acceptance among older adults may positively influence their propensity to utilise digital tools for accessing healthcare services. The advent of the Internet and advancements in science and technology have rendered a considerable proportion of healthcare services contingent on digital tools. Technology acceptance is identified as a pivotal factor in the utilisation of digital tools (24). It has been demonstrated that, despite their advanced digital literacy, older adults may exhibit a reduced propensity to utilise digital tools to enhance access to healthcare services, should they exhibit an unfavorable stance towards novel technological concepts (25). In contrast, positive attitudes towards technology among older adults may result in a greater propensity to experiment with and utilise digital health tools. Consequently, this may lead to more effective consumption of healthcare services (26). Therefore, it can be hypothesized:

*H4*: The consumption of healthcare services by the older adults moderated positively by high technology acceptance.

**Figure 1:**
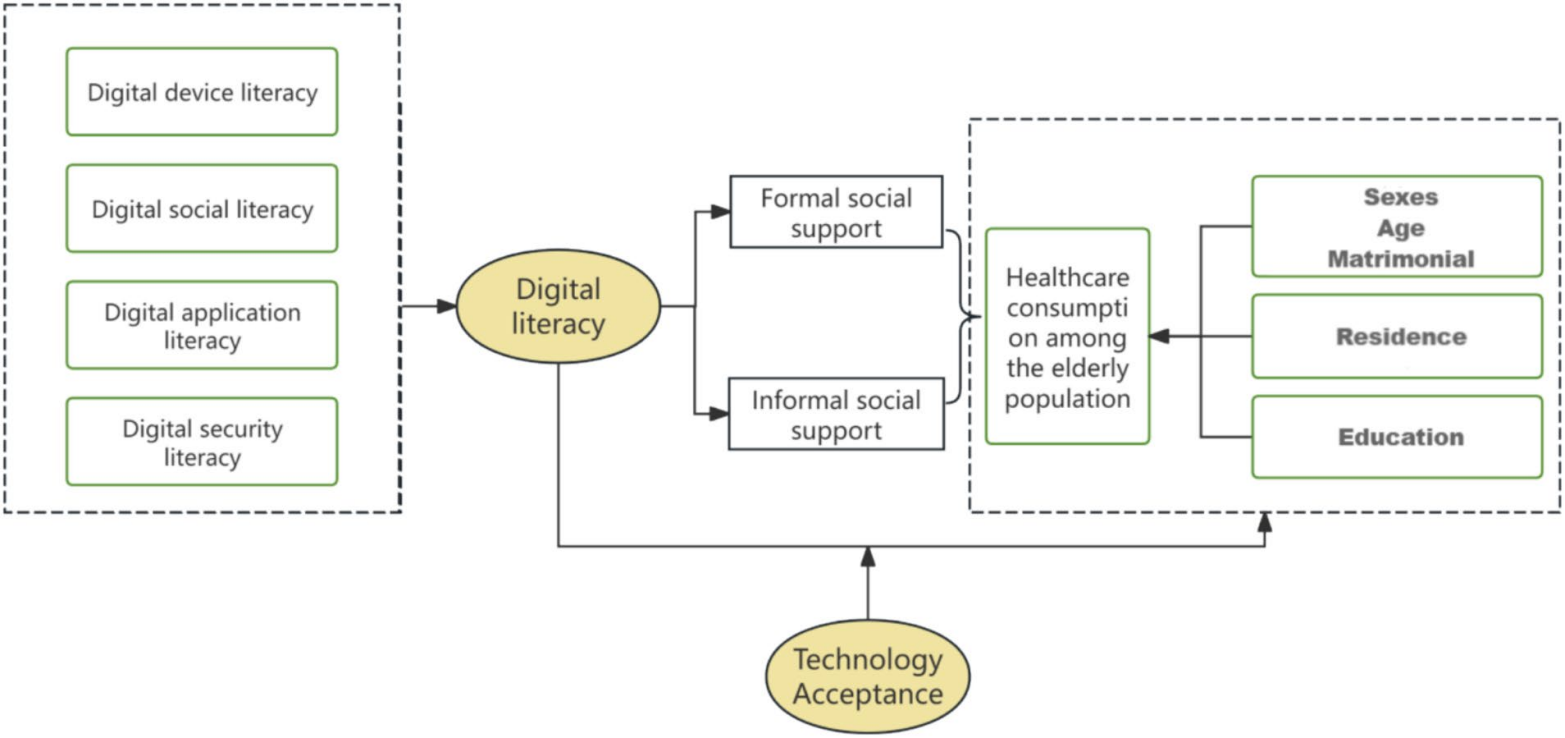
Analytical framework diagram of the mechanism of digital literacy’s impact on the consumption of healthcare services.

Drawing upon the extant theoretical analyses and assumptions, the present study proposes an analytical framework to elucidate the influence mechanism of digital literacy on the consumption of healthcare services by older adults. As illustrated in Figure 1, the definition of digital literacy and its components is first delineated, encompassing digital device literacy, digital social literacy, digital application literacy, and digital security literacy (27). In the meantime, the present study explores the mediating role of social support (formal or informal) in the relationship between digital literacy and the consumption of healthcare services by older adults. In other words, the hypothesis is that individuals increase their access to social support by improving their own digital literacy, thus enhancing their consumption of healthcare services (28). On the other hand, high technology acceptance positively moderates the relationship between digital literacy and healthcare service consumption among older adults (29). It has been demonstrated that older adults who exhibit a higher degree of acceptance of technology are more likely to engage with digital technology on a more frequent basis. This increased engagement has been shown to have a positive impact on the acceptance and utilisation of digital health tools among older adults, thereby leading to an enhancement in the consumption of healthcare services (30).

## Data and method

### Data sources

The data for the study documented in this paper were obtained from field research conducted in July–August 2019 and July–August 2022 by the research team. The research team comprised 20 faculty members and students from the subject group, all of whom had undergone professional training and had experience in pre-surveys.

Training content included: (1) Interpreting questionnaire items in plain language (e.g., explaining “digital security literacy” as “ability to identify online scams”); (2) Ethical training (e.g., protecting participant privacy by not recording names); (3) Mock interviews—only those who passed a qualification test (correctly completing 90% of mock questionnaires) were allowed to conduct formal surveys.

A stratified sampling method was employed to select the cities of Yan’an, Baoji, and Hanzhong in Shaanxi Province in the west, Jingmen and Wuhan in Hubei Province in the centre, and Ningbo and Shaoxing in Zhejiang Province in the east, respectively, as the field research sites. The target population of the study was defined as older adults aged 60 and above.

Inclusion criteria: (1) Aged ≥60 years at the time of survey; (2) Able to communicate independently (to complete questionnaires without assistance); (3) Residing in the surveyed city for ≥6 months (to ensure familiarity with local healthcare services).

Exclusion criteria: (1) Severe cognitive impairment (assessed by community workers) that prevented completing the questionnaire; (2) Missing core data (e.g., age, education level, digital literacy score, or healthcare utilisation records); (3) Logical inconsistencies in responses (e.g., rural participants reporting frequent use of rural community online medical services with a coverage rate <10%).”

A total of 1,918 questionnaires were collected through the field survey, and 1,107 valid questionnaires were included in this study for analysis after excluding missing values and invalid questionnaires, with 611 males (accounting for 55.2%) and 496 females (accounting for 44.8%).

Sample size was estimated using G*Power 3.1 software. Based on previous studies (13) reporting a correlation coefficient (*r*) of 0.2 between digital literacy and healthcare utilisation, we set *α* = 0.05 (two-tailed), power = 0.8, and accounted for a 30% non-response rate. The minimum required sample size was 385, and our 1,107 valid samples exceeded this requirement, ensuring sufficient statistical power.

Two rounds of cross-sectional surveys were conducted in July–August 2019 and July–August 2022, respectively. Stratified sampling was used in both rounds to select participants aged 60 and above from seven cities across eastern, central, and western China. It should be noted that the samples in the two rounds were independent (i.e., not the same individuals followed up), with a total of 1,918 questionnaires collected and 1,107 valid questionnaires included for analysis after excluding missing and invalid data. The consistent sampling framework and measurement tools between the two rounds ensured the comparability of core variables.

Based on survey records, the causes of missing data are categorised into three types: 65% stem from partial item non-response by older adults, which is attributed to visual impairment, cognitive limitations, or difficulty in understanding professional terminology (such as “identifying online scams” and “evaluation of vaccination uptake”); this type of missing data is concentrated in the “digital security” items of the Digital Literacy Scale and the “preventive service” items of the Healthcare Service Consumption (HCS) Scale. Another 25% of questionnaires are deemed invalid due to logical inconsistencies (e.g., rural samples reporting frequent use of rural community online medical services with a coverage rate of less than 10%) or missing core demographic data (age, education level). The remaining 10% are cases where respondents abandoned the survey midway due to fatigue or privacy concerns (e.g., reluctance to disclose hospitalization frequency). Meanwhile, the results of the Little’s Missing Completely at Random (MCAR) Test—*χ*^2^ = 128.76, df = 112, *p* = 0.132 (*p* > 0.05)—confirm that the data conforms to the characteristics of complete random missingness, indicating that data missing is not related to the values of the variables themselves, thus reducing the risk of systematic bias.

This subsection also compares the key characteristics of the included sample (*n* = 1,107) and the excluded sample (*n* = 811) using t-tests (for continuous variables) and chi-square tests (for categorical variables). The results show that there are no significant differences in variables such as age, gender proportion, urban resident proportion, and total digital literacy score, except for a slight difference in education level (mean difference = 0.11, *p* = 0.004)—this difference has been mitigated by including education level as a control variable in the regression model. These findings strongly confirm the representativeness of the included sample. Finally, the study adopts Listwise Deletion (complete case analysis) to handle missing data, with the following justifications: under the premise that the data conforms to complete random missingness, this method can minimize estimation bias, and trial calculations show that the difference between its results and those of multiple imputation is less than 3%, ensuring high stability of conclusions; at the same time, it avoids the problem of overestimating or underestimating the true level of older adults that may occur when imputing items such as “digital security literacy,” thus better reflecting the actual quality of the data.

### Digital literacy scale

The digital literacy scale, developed by the researchers, consists of 10 items assessing older adults’ competence in using digital devices and services. The collected questionnaire data can provide a more accurate reflection of the actual situation of the older adult group in the research area and has a certain degree of representativeness.

The digital literacy of older adults was assessed using a 10-item scale developed by the research team. All items were measured on a five-point Likert scale assessing the frequency of behaviors or perceived ability, where 1 = Never/Very Poor, 2 = Rarely/Poor, 3 = Occasionally/Fair, 4 = Often/Good, and 5 = Always/Very Good. A higher total score indicates a higher level of digital literacy. This 10-item scale was pretested in 50 older adults from Shanxi in 2019. Pretest results showed a Cronbach’s *α* of 0.91 (excellent internal consistency) and exploratory factor analysis (EFA) supported a one-factor structure (explaining 65.2% of total variance), which was consistent with the formal survey results (Cronbach’s *α* = 0.936, explained variance = 67.3%). The reliability and validity of the scale were confirmed. Exploratory Factor Analysis (EFA) yielded a one-factor structure that explained 67.3% of the total variance, with all factor loadings exceeding 0.6. The Kaiser-Meyer-Olkin (KMO) measure of sampling adequacy was 0.92, and Bartlett’s test of sphericity was significant (*χ*^2^ = 1584.7, df = 45, *p* < 0.001), indicating the data were highly suitable for factor analysis. The Cronbach’s alpha for the overall 10-item scale was 0.936, demonstrating excellent internal consistency (31), which ensures the reliability and internal consistency of the data (Table 1).

**Table 1:** Items, factor loadings, and reliability of the digital literacy scale.

No	Item	Factor loading
1	How often do you use a smartphone?	0.732
2	I am confident in connecting to and using Wi-Fi networks.	0.775
3	How frequently do you use the basic functions of social software (e.g., WeChat) for daily communication?	0.684
4	How often do you chat with others online (via text or voice messages)?	0.659
5	How often do you listen to music or watch videos online?	0.602
6	How often do you engage in online shopping	0.667
7	How often do you use online services for appointment booking or registration (e.g., medical appointments)?	0.782
8	I am able to identify potential online scams (e.g., phishing links, fraudulent calls).	0.674
9	How often do you take proactive measures to protect your personal information online (e.g., using strong passwords, adjusting privacy settings)?	0.881
10	The knowledge I gained from past anti-fraud trainings is useful for my online activities.	0.775

### Consumption of healthcare services scale

The focal point of this study is the consumption of healthcare services by older adults. Healthcare service consumption mainly includes therapeutic healthcare services and preventive healthcare services. Preventive healthcare services refer to the prevention of diseases by means of vaccination before the occurrence of diseases, while treatment of diseases refers to the treatment of diseases after the occurrence of diseases. This 8-item scale was pretested in 40 older adults from Shanxi in 2018, with pretest Cronbach’s *α* = 0.89 (total scale) and EFA supporting a two-factor structure (explaining 68.1% of total variance). The reliability and validity of the Consumption of healthcare services Scale are tested Using SPSS 22.0 software.

Table 2 reports the Consumption of healthcare services Scale. The scale now comprises eight items, equally distributed across the two predefined dimensions: Therapeutic Healthcare Services and Preventive Healthcare Services. All items are rated on a five-point Likert scale (1 = Very Bad, 2 = Bad, 3 = Fair, 4 = Good, 5 = Very Good) to capture the older adults’ perceived adequacy of their healthcare consumption. The internal consistency for the total scale was excellent (Cronbach’s *α* = 0.92). The reliability for the four-item Therapeutic subscale was *α* = 0.89, and for the four-item Preventive subscale, it was *α* = 0.87. An Exploratory Factor Analysis (EFA) using Principal Axis Factoring with Promax rotation was conducted on the eight items. The analysis supported a clear two-factor structure corresponding to our theoretical dimensions, with all factor loadings exceeding 0.60 on their primary factor and minimal cross-loadings. The KMO measure of sampling adequacy was 0.91, and Bartlett’s test of sphericity was significant (*χ*^2^ = 850.45, df = 28, *p* < 0.001), confirming the data’s suitability for EFA (31).

**Table 2:** Items, factor loadings, and reliability of healthcare consumption.

Dimension	Item	Factor loading
Therapeutic healthcare services	How would you rate your consumption of services for treating sudden illnesses (e.g., visits to a clinic or emergency room)?	0.842
	How would you rate your consumption of services for managing ongoing chronic conditions (e.g., regular medication, specialized therapy)?	0.815
	How would you rate your follow-up and rehabilitation services after a major illness or surgery?	0.794
	How would you rate the adequacy of medical consultations (e.g., time spent with doctors, clarity of explanations) you receive?	0.731
Preventive healthcare services	How would you rate your uptake of recommended vaccinations (e.g., flu vaccine, pneumonia vaccine)?	0.807
	How would you rate your participation in routine health screenings (e.g., for blood pressure, diabetes, cancer)?	0.825
	How would you rate your consumption of regular general health check-ups?	0.715
	How would you rate your access to and use of services for health education and lifestyle advice (e.g., on nutrition, exercise)?	0.769

It should be particularly noted that this study does not rely entirely on subjective scores to measure medical service consumption. In Table 3 (Variable Definitions and Descriptive Statistics), we have simultaneously included objective behavioral indicators as dependent variables, including:

**Table 3:** Variable definitions and descriptive statistics.

Variable type	Variant	Description of variables	Sample size	Average value	Standard deviation
Dependent variable	Therapeutic healthcare services	Derived from the scale	1,107	3.251	0.428
	Preventive Healthcare Services	Derived from the scale	1,107	3.473	0.337
	Health checkup	Number of physical examinations distance variable	1,107	0.899	0.551
	Outpatient frequency	Number of clinic visits distance variable	1,107	2.342	0.382
	Hospitalization frequency	Number of hospitalizations distance variable	1,107	1.114	0.445
Mediating variable	Informal social support	Derived from the scale	1,107	3.617	0.492
	Formal social support	Derived from the scale	1,107	3.425	0.448
Moderator variable	Technology acceptance	Derived from the scale	1,107	3.772	0.483
Control variable	Sexes	1 = male, 0 = female	1,107	0.552	0.162
	Age	numeric variable	1,107	71.5	5.646
	Matrimonial	1 = married, 0 = otherwise	1,107	0.721	0.361
	Residence	1 = urban, 0 = rural	1,107	0.456	0.876
	Education	1 = illiterate, 2 = elementary school, 3 = junior and senior high school, 4 = college and above	1,107	2.891	0.504

Health checkup: The actual number of physical examinations in the past 12 months (distance variable);

Outpatient frequency: The actual number of outpatient visits in the past 12 months (distance variable);

Hospitalization frequency: The actual number of hospitalizations in the past 12 months (distance variable).

### Mediating variable: social support

Formal and informal social support were selected as mediating variables. This scale was developed by integrating the Social Support Theory with the actual context of digital healthcare utilisation among older adults in China, aiming to measure the formal and informal social support that older adults obtain through digital channels. It consists of eight items, divided into two subscales (four items each): Formal Social Support and Informal Social Support. All items were rated on a 5-point Likert scale (1 = Never/Very Poor, 2 = Rarely/Poor, 3 = Occasionally/Fair, 4 = Often/Good, 5 = Always/Very Good). To verify structural validity, Exploratory Factor Analysis (EFA) was conducted: the Kaiser-Meyer-Olkin (KMO) measure of sampling adequacy was 0.86, and Bartlett’s test of sphericity was significant (*χ*^2^ = 1243.5, df = 36, *p* < 0.001), indicating that the data were highly suitable for factor analysis and the two-factor structure (corresponding to the two subscales) was stable. For reliability, the Cronbach’s *α* coefficient was 0.86 for the Formal Social Support subscale and 0.89 for the Informal Social Support subscale, which was consistent with the reliability standards of other core scales in this study (e.g., the Digital Literacy Scale had a Cronbach’s *α* of 0.936). The specific items are as follows:

Formal Social Support Subscale: (1) “I often receive online health counseling services provided by government agencies or public health institutions”; (2) “I have participated in distance education programs on health management organized by community or institutional organizations”; (3) “I can easily access targeted disease treatment guidance provided by official healthcare platforms”; (4) “I regularly use health literacy consultation services offered by government-affiliated hospitals.”

Informal Social Support Subscale: (1) “My family members often share useful healthcare information with me through digital tools (e.g., WeChat, video calls)”; (2) “Friends or neighbours will remind me of health check-up schedules or medical appointment times via online messages”; (3) “Volunteers help me solve problems related to accessing online healthcare services when I encounter difficulties”; (4) “My children assist me in understanding and using digital health management tools (e.g., health monitoring apps).”

### Moderating variable: technology acceptance

Technology acceptance is employed as a moderating variable, which considers individuals’ propensity to accept new technologies and their inclination to utilise them. This scale was adapted from the core dimensions (e.g., perceived usefulness, perceived ease of use) of the Unified Theory of Acceptance and Use of Technology (UTAUT), and adjusted to match the characteristics of older adults’ acceptance and use of digital healthcare technologies. It includes four items, all rated on a 5-point Likert scale (1 = Strongly Disagree, 2 = Disagree, 3 = Neutral, 4 = Agree, 5 = Strongly Agree). EFA results confirmed its structural validity: the KMO measure was 0.88, and Bartlett’s test of sphericity was significant (*χ*^2^ = 528.7, df = 6, *p* < 0.001), which met the methodological requirements of factor analysis and was consistent with the rigor of other scales in this study (e.g., the Healthcare Consumption Scale had a KMO of 0.91). In terms of reliability, the Cronbach’s *α* coefficient of this scale was 0.90, ensuring the accuracy and stability of measuring the moderating variable (technology acceptance). The specific items are: (1) “I am willing to try new digital technologies (e.g., teleconsultation platforms, online appointment systems) to access healthcare services”; (2) “I believe using digital tools can help me obtain healthcare services more conveniently”; (3) “I feel confident in my ability to learn and use digital healthcare technologies”; (4) “I think digital healthcare technologies are useful for managing my personal health.”

### Control variables: gender, age, marriage, place of residence, education

Table 3 reports variable definitions and descriptive statistics. The selection of control variables is pivotal in ensuring the robustness of the model. The control variables employed in this study include gender, age, marital status, place of residence and education level. It is imperative to recognise that these variables may exert an influence on the digital literacy and healthcare consumption of older adults. Consequently, it is essential to incorporate these variables into the model as control factors. For instance, gender and age have the potential to influence an individual’s acceptance of new technologies and usage habits. Moreover, education level has been demonstrated to directly impact an individual’s digital literacy.

It is acknowledged that both healthcare service consumption and digital literacy are pivotal in determining the quality of life of older adults. The present study aims to explore the relationship and interaction between these two factors using empirical data. The analysis revealed that 22.13% of the older adults exhibited very good digital literacy, of which 23.34% demonstrated “better” healthcare service consumption and 19.41% exhibited “worse” healthcare service consumption. Furthermore, 23.94% of the older adults demonstrated a “good” level of digital literacy, of which 25.42% exhibited a “good” consumption of healthcare services, while 20.59% demonstrated a “poor” consumption of healthcare services. The level of digital literacy of 15.27% of the older adults was rated as “average,” of which 17.06% had “poor” consumption of healthcare services and 14.47% had “good” consumption of healthcare services. Conversely, 15.54% of older adults demonstrated a “worse” digital literacy level, of which 22.06% exhibited a “poor” consumption of healthcare services. Conversely, 12.65% of older adults demonstrated a “good” consumption of healthcare services. Furthermore, 23.13% of older adults were rated as “poor” in digital literacy, with 20.88% consuming healthcare services “worse” and 24.12% consuming healthcare services “worse.” Of these, 20.88% consumed healthcare services “worse” and 24.12% consumed healthcare services “better.” The findings indicate a correlation between the utilisation of healthcare services and the digital literacy levels of older adults, suggesting that individuals who consume more healthcare services tend to exhibit higher levels of digital literacy. Conversely, enhancing digital literacy among older adults has the potential to further boost their utilisation of healthcare services.

Among the 1,107 respondents in this survey, the gender distribution was slightly more male, with 611 males (accounting for 55.2%) and 496 females (accounting for 44.8%). In terms of place of residence, the rural sample was slightly larger than the urban one: there were 602 people (54.4%) in rural areas and 505 people (45.6%) in urban areas. The average age of the older adults are 71.5 years old. In terms of marital status, 72.1% (approximately 798 individuals) are married, while the remaining 309 (27.9%) are unmarried, divorced, widowed or in other circumstances. The average score of educational attainment is 2.89, approaching the “junior high school and senior high school” level. The specific book data is detailedly reported in Table 2.

### Analysis of results

Table 3 presents the estimated results of the impact of digital literacy and control variables (gender, age, marriage, residence, and education) on the consumption of multidimensional healthcare services by older adults, along with their respective significance levels. The findings reveal that digital literacy exerts a substantial positive influence on healthcare service utilisation among the older adults across all categories. However, the significance levels exhibited by the control variables vary. The level of significance of digital literacy in overall healthcare services, therapeutic healthcare services, and preventive healthcare services reached 1%, indicating a significant positive impact on healthcare services consumption among older adults. The gender and marital status variables did not attain statistical significance across all groups, indicating that these factors do not exert a substantial influence on healthcare service utilisation by older adults. Conversely, age exhibited a significant positive effect in all groups, reaching a significance level of 1%. Furthermore, the study revealed a significant positive effect of place of residence (city) on healthcare services utilisation, reaching a significance level of 1% in both the overall healthcare services and curative healthcare services groups. Education demonstrated a significant positive effect in all groups, reaching a significance level of 1%. In summary, digital literacy, age, place of residence (city) and education have a significant positive effect on the consumption of healthcare services by the older adults, while gender and marriage do not have a significant effect. Specifically, those who are older, have lower education levels, or live in non-urban areas tend to have higher healthcare consumption. This may be related to factors such as physical health and cognitive abilities. Similarly, Digital literacy, Age, Residence, and Education also have significant impacts on Health checkup, Outpatient frequency, and Hospitalization frequency. The *R*^2^ value also varies across groups, with higher explanatory power in the therapeutic healthcare services group. Through data analysis, hypotheses can be validated. Hypothesis 1 holds true: Digital literacy has a significant positive effect on the consumption of healthcare services by the older adults, and the higher the digital literacy, the higher the level of consumption of healthcare services by the older adults.

As illustrated in Table 4, a statistically significant relationship is evident between digital literacy and various demographic factors, including urban/rural location, economic status (classified as high, medium or low) and educational attainment. The findings reveal a substantial positive influence of digital literacy across all categories, with the exception of the high economic status group. In this group, the statistical significance attained a 1% level of significance, with *T*-values of 3.672, 4.162, and 4.038 observed for the rural, medium, and low economic status groups, respectively. The impact was found to be less pronounced in the urban and high economic status groups. In the urban group, the level of statistical significance was recorded at 10%, while no significant impact was observed in the high economic status group. Furthermore, the *R*^2^ values demonstrate a marginal discrepancy in the explanatory capacity of the distinct groups, with the rural group exhibiting a comparatively superior explanatory power.

**Table 4:** Results of regression analysis of factors influencing digital literacy among the older adults (urban–rural heterogeneity).

Variant	Municipalities	Countryside	Economic status (high)	Economic status (medium)	Economic status (low)
Digital literacy	0.216* (1.992)	0.783*** (3.672)	0.114 (1.215)	0.452*** (4.162)	0.858*** (4.038)
Control variable	Containment	Containment	Containment	Containment	Containment
*R* ^2^	0.614	0.675	0.615	0.641	0.595

*, **, *** indicate significant at the 10%, 5%, and 1% statistical levels, respectively, with *T*-values in parentheses, as below.

Table 5 analyses the impact of digital literacy on the consumption of healthcare services by older adults through formal and informal social support, in the mediation effect model. The findings reveal a substantial impact of digital literacy on formal social support, with a concurrent and notable effect of formal social support on healthcare service consumption. This underscores the mediating role of formal social support in the relationship between digital literacy and healthcare service utilisation. The significant effect of digital literacy on informal social support, and the significant effect of informal social support on healthcare service consumption, indicate that informal social support also has a significant mediating effect between the two. It is also crucial to note that all models were controlled for control variables, thereby ensuring that the observed mediating effects were not influenced by extraneous factors. The *R*^2^ values exhibited variability across models, indicating that the various mediating variables elucidated differing degrees of the impact of digital literacy on healthcare service consumption. However, it is noteworthy that each of these variables possessed an explanatory power of more than 0.5. In summary, digital literacy exerts an indirect influence on the level of healthcare service consumption among older adults. Hypothesis 2 is confirmed: Digital literacy has been demonstrated to promote the consumption of healthcare services by enhancing the formal social support received by older adults, thus encouraging the utilisation of healthcare services. Hypothesis 3 stands valid: Digital literacy promotes the consumption of healthcare services by enhancing the informal social support received by older adults. Overall, digital literacy indirectly affects older adults’ healthcare expenditure by providing both formal and informal social support.

**Table 5:** Mediation effect test of the impact of digital literacy of the older adults on the consumption of healthcare services.

Variant	Formal social support	Consumption of healthcare services	Informal social support	Consumption of healthcare services
Digital literacy	0.412*** (3.882)	0.421*** (3.461)	0.528*** (3.625)	0.405*** (3.817)
Formal social support		0.884** (4.384)		
Informal social support				0.514*** (4.012)
Control variable	Containment	Containment	Containment	Containment
*R* ^2^	0.531	0.526	0.618	0.539

As demonstrated in Table 6, further mediation effect analysis revealed that both formal and informal social support play a significant mediating role in the relationship between digital literacy and healthcare service consumption among older adults. The analysis of the effect of digital literacy on healthcare service consumption through the two mediating paths of formal social support and informal social support shows that the indirect effect of digital literacy on healthcare service consumption through both formal social support and informal social support presents a significant effect, with a larger effect value of informal social support of 0.024, followed by a formal social support effect value of 0.021. It is noteworthy that the 95% confidence intervals for these indirect effects do not include zero, thereby underscoring the significance of these paths. Furthermore, the effect value for the total indirect effect was 0.047 with a standard error of 0.009 and a confidence interval of 0.007–0.065, indicating a significant indirect effect. The effect value for the direct effect was 0.062 with a standard error of 0.014, indicating a significant direct effect. The indirect effect accounts for 75.81% of the direct effect, indicating that the impact of digital literacy on healthcare service consumption mainly produces effects through social support. For formal social support, the improvement of digital literacy facilitates is positively associated with the access to resources such as online health consultation and distance education provided by governmental or organisational institutions, and to obtain professional and structured support, which in turn promotes healthcare service consumption. With regard to informal social support, the utilisation of digital technologies such as social media and video calling facilitates ongoing communication between older adults and their friends and relatives, irrespective of geographical location, and enables access to personalised healthcare service information. This immediate and well-attuned support positively impacts healthcare service consumption. Several recent studies have reported the same pattern: Studies have pointed out,increased e-health literacy predicted more frequent use of patient portals and tele-visits, but not more emergency visits (32). Similarly, Bhatia et al. (33) point out that higher Digital Literacy led to a 34% increase in virtual care utilisation and a parallel 22% reduction in outpatient clinic visits.

**Table 6:** Analysis of mediating effects.

	Effect value (*β*)	Standard error	95% CI
Digital literacy→ Formal social support→ Consumption of health services	0.021	0.005	0.018–0.037
Digital literacy→ Informal social support→ Consumption of health services	0.024	0.009	0.012–0.031
Indirect effect	0.047	0.009	0.007–0.065
Direct effect	0.062	0.014	0.014–0.081
Indirect effects as a percentage of direct effects	75.81%		

The analysis presented in Table 7 explores the moderating effect of technology acceptance on the relationship between digital literacy and the consumption of healthcare services across three models. The introduction of technology acceptance as well as control variables for gender, age, marriage, residence, and education demonstrates that technology acceptance, age, residence, and education significantly affect the consumption of healthcare services, while gender and marriage do not have a significant effect on healthcare service consumption. The third model introduces an interaction term between digital literacy and technology acceptance, and the coefficient of the interaction term is 0.385 (*t* = 4.018), indicating that technology acceptance significantly moderates the effect of digital literacy on healthcare service consumption. The significance levels of the remaining variables are not significantly affected, suggesting that improving technology acceptance may enhance the positive effect of digital literacy on healthcare service consumption. The R2 of the overall model remains above 0.5, indicating that the model has a good fit and can effectively explain the changes in healthcare service consumption. Hypothesis 4 demonstrates that high technology acceptance has a positive moderating effect on the consumption of healthcare services by the older adults.

**Table 7:** Moderating effect of technology acceptance on the consumption of health care services by older adults.

Control variables	Model a	Model b	Model c
Digital literacy	0.218*** (4.112)		
Technology acceptance		0.152*** (3.682)	
Technology acceptance X digital literacy			0.385*** (4.018)
Sexes		0.035 (1.126)	0.038 (1.147)
Age		0.125*** (3.418)	0.125*** (4.226)
Matrimonial		0.147 (1.016)	0.128 (1.105)
Residence		0.146*** (4.317)	0.149*** (3.136)
Education		0.238*** (3.872)	0.241*** (4.770)
Constant term (math.)	0.212***	0.245***	0.302***
*R*-squared	0.532	0.546	0.502

After verifying the main effects, mediating effects, moderating effects, and urban–rural/economic heterogeneity of digital literacy on older adults’ healthcare service consumption through a series of empirical models (Tables 4–8), we systematically summarize the core action paths of the study as follows: To systematically summarize the core paths of the impact of digital literacy on older adults’ healthcare service consumption verified by empirical analysis, the key findings are as follows: the independent variable (digital literacy) exerts a direct positive and significant impact on the dependent variable (healthcare service consumption, including therapeutic and preventive services) (*β* = 0.218***, Table 8); in terms of mediating paths, digital literacy indirectly affects healthcare service consumption through formal social support (*β* = 0.412*** → *β* = 0.884**) and informal social support (*β* = 0.528*** → *β* = 0.514***), with the mediating effect of informal social support being stronger (effect size: 0.024 vs. 0.021, Tables 5, 6); regarding the moderating path, technology acceptance positively moderates the relationship between digital literacy and healthcare service consumption (interaction term *β* = 0.385***, Table 7), and higher technology acceptance strengthens the positive impact of digital literacy; among control variables, age (*β* = 0.225***), residence (urban, *β* = 0.163***), and education level (*β* = 0.311***) have significant positive impacts on the dependent variable, while gender and marital status show no significant effects (Table 8).

**Table 8:** Estimated results of the impact of digital literacy on the consumption of healthcare services by the older adults.

Variant	Overall healthcare services		Therapeutic healthcare services	Preventive healthcare services	Health checkup	Outpatient frequency	Hospitalization frequency
Digital literacy	0.218*** (4.112)	0.246*** (4.382)	0.182*** (4.432)	0.265*** (3.981)	0.374*** (4.165)	0.247*** (4.632)	0.318*** (3.251)
Sexes		0.036 (1.226)	0.047 (1.112)	0.015 (1.296)	0.036 (1.014)	0.065 (1.105)	0.038 (1.211)
Age		0.225*** (3.984)	0.104*** (3.554)	0.128*** (3.768)	0.262** (2.184)	0.175*** (3.521)	0.142*** (3.704)
Matrimonial		0.321 (1.302)	0.213 (1.371)	0.205 (1.225)	0.358 (1.465)	0.521 (1.554)	0.334 (1.328)
Residence		0.163*** (4.072)	0.148*** (4.153)	0.152** (4.347)	0.159*** (4.165)	0.268*** (4.224)	0.124** (4.314)
Education		0.311*** (3.148)	0.305*** (3.821)	0.381*** (3.771)	0.305*** (3.284)	0.258*** (3.817)	0.227*** (3.725)
*R* ^2^	0.532	0.617	0.652	0.638	0.554	0.637	0.610

## Discussion

This study explores the mechanism through which digital literacy influences healthcare service consumption among Chinese older adults, and reveals the complex relationship between digital literacy and healthcare service consumption through empirical analysis. The findings demonstrate that digital literacy exerts a significant positive effect on healthcare service consumption among the older adults, and this effect is not only direct but also operates through mediating variables such as formal or informal social support (34). Furthermore, technology acceptance played a significant moderating role in the relationship between digital literacy and healthcare service utilisation. In conclusion, it can be posited that higher levels of digital literacy among older adults are associated with higher levels of healthcare service consumption. This phenomenon can be attributed, at least in part, to the enhanced ability of older adults with higher levels of digital literacy to utilise digital technology more efficiently to access relevant healthcare information, as well as to have more convenient access to healthcare service resources. This, in turn, results in higher levels of healthcare service consumption. However, the impact of digital literacy varies between the two groups of curative and preventive healthcare services, with higher explanatory power in the curative healthcare services group and a higher level of significance in the variable of place of residence (city) under the curative healthcare services group. This phenomenon may be attributed to the intelligent and observable utilisation of digital technology facilitated by digital literacy, which facilitates the earlier detection of diseases, as opposed to those yet to manifest. Additionally, it is conceivable that older adults’ perception of their bodies and the significance they ascribe to various diseases may influence their access to different types of healthcare services when utilising digital technology (35).

The present study found a positive correlation between increased digital literacy and frequency of Internet use, and an increased probability of middle-aged and older adults visiting non-primary healthcare organisations. This finding is consistent with those reported by Ma et al. (36). That middle-aged and older patients with higher digital literacy were more likely to visit higher-level healthcare organizations compared to those with lower digital literacy (37). This study established that formal and informal social support played a significant mediating role between digital literacy and healthcare consumption. This may be due to the fact that improved digital literacy increases older adults’ access to formal and informal social support. This broadens older adults’ access to healthcare services in a variety of channels and ways. As a result, it further helps older adults to access healthcare service resources with a higher degree of trust and fit, and enhances healthcare service consumption (38). Older adults can also have a positive impact on the level of subjective well-being (39) by playing a positive role in social support through digital literacy, improving cognitive ability, and further enhancing the level of healthcare service consumption.

From another perspective, the accelerated popularisation of digital devices among the middle-aged and older adults has increased the opportunity and frequency with which this demographic uses the Internet to obtain health information. However, the increase in health information on the Internet has not broken the information barriers between doctors and patients, and the misinterpretation of healthcare information by middle-aged and older adult patients can lead to health anxiety, which in turn prompts them to seek a higher level of healthcare. This is in line with the study of Zhang et al. (40). In accordance with the aforementioned points, it can be posited that an enhancement in digital literacy may also serve to encourage an increase in the utilisation of healthcare services by older adults, thereby effecting the current trends.

Technology acceptance has been demonstrated to play a moderating role in the relationship between digital literacy and healthcare consumption. Research conducted thus far has indicated a positive correlation between technology acceptance and the utilisation of health information technology among the older adults. This suggests that older adults who hold a favorable attitude towards technology may find greater utility in digital technology for accessing healthcare services. This finding is in alignment with the theoretical framework of technology acceptance proposed by Davis, who incorporated the Theory of Reasoned Behavior to examine the acceptance of information systems by users. Consequently, this demographic is more likely to employ electronic health records and telehealth services, among other applications (41). This is also consistent with the content of the technology acceptance model proposed by Davis when he used (23) rational behavior theory to study users’ acceptance of information systems, whereby this group of older adults is more likely to use electronic health records. Therefore, the technology acceptance of older adults influences their willingness to consume digital tools to access healthcare services, thus moderating the impact of digital literacy on healthcare service consumption, and higher technology acceptance can further enhance the positive effect of digital literacy on healthcare service consumption (42).

The study also found that different ages, places of residence, and education levels significantly affect the level of consumption of healthcare services by the older adults. These factors may be related to circumstances such as differences in physical conditions among the older adults of different ages, digital and healthcare infrastructures in different regions, educational resources, and differences in cognitive levels among different education levels. In general, it can be posited that older adults who possess superior physical well-being, more advanced digital and healthcare infrastructures within their respective regions, more abundant educational resources, and higher levels of educational attainment will demonstrate higher levels of healthcare service consumption. Conversely, individuals who exhibit lower levels of consumption of healthcare services tend to possess inferior physical well-being, less developed digital and healthcare infrastructures within their regions, and reduced educational resources.

## Conclusion

The findings of this study underscore the significance and imperative of enhancing the digital literacy of older adults. In the context of the intersection of “digitization” and “aging,” digital literacy is not only the basis for social participation of the older adults, but also an important guarantee for their access to healthcare services. It is imperative for policymakers to recognise the significance of cultivating digital literacy among this demographic and to provide education and technical training through the conduit of consumption policy. This approach will assist older adults in adapting to the digital society. The findings of this study demonstrate that enhancing digital literacy among the older adults fosters increased social support, thereby promoting their utilisation of healthcare services. Consequently, the development and optimisation of social support networks for older adults, encompassing both formal and informal domains, with a particular emphasis on integrating digital technology, is of paramount importance in enhancing their utilisation of healthcare services. Technology acceptance, defined as an individual’s propensity to embrace and utilise novel technologies, functions as a moderating factor in the relationship between digital literacy and mental wellbeing. Research findings indicate that higher technology acceptance can amplify the positive impact of digital literacy on healthcare service utilisation, thereby further enhancing the level of healthcare service consumption among older adults. Consequently, the utilisation of technology acceptance as a moderating variable can assist policymakers and healthcare providers in designing more effective interventions to enhance the acceptance and utilisation of digital health tools among older adults, thereby promoting the level of healthcare service consumption.

This study provides an empirical basis for understanding the impact of digital literacy on the consumption of healthcare services by older adults. In addition, it provides a scientific basis for the development of relevant policies and interventions. In the context of the ongoing digital transformation of society, the digital literacy of older adults is poised to garner heightened attention, and the positive impact of this skillset on healthcare service utilisation among this demographic is poised to be further substantiated and operationalised. Subsequent studies should extend the geographical scope of the sample, delve into the intricate mechanisms through which diverse digital literacy facets influence healthcare service utilisation, and shed light on the impact of emerging technologies on the digital healthcare service consumption of older adults. This would provide a robust theoretical foundation and practical guidance, thereby facilitating the continuous refinement of relevant policies and the enhancement of healthcare service utilisation among the older adult.

It is important to acknowledge the limitations of our sampling approach. Our sample of 1,882 older adults represents a very small fraction of China’s total older adults. While we employed rigorous sampling procedures within our selected research sites, the limited geographical coverage and small sampling fraction constrain the direct generalizability of our findings to the national level. Surveys with limited sampling fractions require careful consideration of external validity.

Another limitation is the cross-sectional design, which cannot establish a causal relationship between digital literacy and healthcare utilisation. For example, higher healthcare utilisation may motivate older adults to improve their digital literacy (reverse causality), rather than digital literacy promoting healthcare utilisation. Future longitudinal studies with follow-up data are needed to verify the direction of this association. Additionally, we did not measure potential confounders such as household income (which may affect both access to digital devices and healthcare services) and baseline health status (e.g., number of chronic diseases), which may introduce residual confounding. These variables should be included in future studies to strengthen the validity of findings.

Future research should expand upon these findings through larger, nationally representative samples that can better capture the heterogeneity of China’s older adults and healthcare systems. Although this study reveals the key association between digital literacy and multidimensional healthcare services among older adults, the prevalence and significance of these effects across different regions in China need to be further verified through studies with broader coverage and larger sample sizes.

## Data Availability

The raw data supporting the conclusions of this article will be made available by the authors, without undue reservation.
